# On Extraction of Soft Cataract by Suction

**Published:** 1865-02

**Authors:** T. Pridgin Teale

**Affiliations:** Surgeon to the Leed's General Infirmary.


*On Extraction of Soft Cataract by Suction.* By T. Pridgin Teale, Jr., Surgeon to the Leed's General Infirmary.

During the autumn of last year I was led to inquire whether the principle of suction might not be made use of in withdrawing from the eye through a small wound all such cataracts, whether a traumatic or spontaneous, as have neither a hard nucleus from old age nor have undergone calcareous degeneration. This inquiry was suggested to me by the difficulty sometimes met with in certain cases of "linear extraction," in which the soft matter is removed either incompletely or only after prolonged and repeated introduction of the curette. I believed, moreover, that if the posterior capsule could be thoroughly cleansed from all opaque matter, without undue violence to the eye, there would be much less risk of inflammatory mischief after linear extraction, and that one of the chief sources of opacity of the posterior capsule would be removed. Having therefore satisfied myself by experiment that the softer portions of even a healthy lens, could be sucked through a fine tube, I requested M. Weiss to make for me a suction instrument which, being modeled upon the ordinary curette, I named a "suction curette."

As I have now had many opportunities of testing the value of extraction by suction, and as since my introduction, or rather, as it proves, revival of the principle, it is being extensively adopted in ophthalmic practice, I venture to offer the following remarks on the mode of performing the operation, and other points of interest connected with it:

*Details of the Operation.*—The operation by suction which I have adopted, is founded upon, and is essentially a modification of "linear extraction," the principles of which have been worked out by Mr. Bowman, and have been clearly and fully described by Mr. Critchett and Mr. Geo. Lawson.

First Stage.—*The efficient Rupture of the Anterior Capsule.*—The pupil having been dilated by atropine, and the eye-lids fixed by the stop-wire speculum, the anterior capsule of the lens should be very freely torn open by two needles passed through the cornea from opposite sides. In carrying out this step the surgeon should bear in mind that its object is not merely to liberate the cataract, but also to insure such a tearing up of the anterior capsule that it may curl back from the area of the pupil and be lodged behind the iris. At the same time, he ought, by all means, to avoid injuring the *posterior capsule*—a caution to be especially remembered in cases where the cataract is dwarfed and the anterior capsule tough, or when, in traumatic cataract, the lens has been much reduced in bulk by absorption. If the operator wishes to avoid the use of the two needles, he may rupture the *capsule* at a later stage by introducing through the corneal opening made for the curette the hook used in extraction of hard cataract. The two needles, however give more perfect command over this important step in the operation.

Second Stage.—*The Opening in the Cornea.*—Having withdrawn one needle, and steadying the eye by means of the other, the operator should next make an opening in the cornea for the admission of the tubular curette of the suction instrument. For this purpose, a broad needle has been made for me by M. Weiss, of such a breadth as to make an opening of the exact size required for the curette.—The needle should enter the cornea opposite the margin of the pupil when fully dilated, and passing somewhat obliquely through the laminæ of the cornea, should make a valvular opening, in order, firstly, that it may not be too central and leave a scar in front of the pupil; secondly, that it may not be too near the attacked margin of the iris, and thus favour its prolapse and adhesion to the wound and thirdly, that the curette, when introduced, may not rest upon nor bruise the iris.

Third Stage.—*The Removal of the Cataract by Suction.*—Having carefully introduced the curette, (if it hitches in traversing the corneal wound, it may easily be disengaged by being turned edgeways,) the surgeon should hold the open end of the tube steadily within the area of the pupil, gently burying it in the opaque material. The suction power may then be applied and regulated in degree as the opaque matter runs off in the tube. As soon as the pupil is clear, the curette may be carefully depressed towards the posterior capsule, in order to ascertain whether any opaque matter remains, but it should not on any account be swept before or behind the iris. If the suction be continued after the opaque matter has been removed, the cornea is drawn down over the open end of the curette and blocks it up, thus preventing the iris from being sucked into the instrument and injured.

If the operation has been efficiently performed, it will be found that the cataract has been completely withdrawn from the eye through an opening in the cornea no larger than would admit the common curette, without any injury to the iris, without rupture of the posterior capsule, and with such complete division of the anterior capsule that it has disappeared completely behind the iris.—It will be found, I think, in the majority of such cases, that recovery is most speedy, that the operation is followed by little or no irritation of the eye, that the patient on the eighth or tenth day can read No. 1, (Jager,) and that the conditions which usually produce opacity of the capsule have been provided against.

The foregoing rules apply to a simple case of complete soft cataract. They are also applicable, with slight modification, to cases of traumatic cataract of recent occurrence. In these cases, however, it is necessary, in the first place, to be very careful to tear open completely the anterior capsule which may have been previously ruptured in the accident producing the cataract; and in the second place, to bear in mind that the posterior capsule may also have been torn through. Should this have occurred, the suction operation will be complicated by the admission into the anterior parts of the eye of the vitreous humour, which would tend to pass through the tube more readily than the denser material of the cataract. When such a defect occurs, it is sometimes possible, by careful management of the curette, to withdraw the opaque lens, without, at the same time, drawing off a serious amount of vitreous humour.

Another complication may arise, namely—partial cataract, in which the nuclear portions of the cataract are opaque, and the cortical portions are healthy, tenaceous, and adherent to the capsule. This difficulty must be met in the same way as in "linear extraction," by the preliminary operation of puncturing the anterior capsule so as to admit the aqueous humour into the structure of the lens, and so to cause its disintegration. It may be possible to withdraw by suction even a partially sound lens without the preliminary disintegration; but I have not yet attempted to do so, not from any difficulty in drawing such healthy lens through the tubular curette, but because when the peripheral parts of the lens are transparent and adhere to the capsule, it is hardly possible to ascertain when the capsule has been completely cleansed from lenticular matter.

Another class of cases presenting difficulties is that in which a soft cataract has become wasted and calcareous, or partly so. In such cases the solid portions will not pass along the tube of the curette.

*On former attempts to extract Cataracts by Suction, and their failure.*—When I first devised the suction-curette, I was under the impression that I had hit upon a new idea, and that the proposal was original. By the kindness of several friends, however, I have been directed to accounts of various previous attempts to apply the same principle to the extraction of cataract.

The Persians, years ago, are said by Avicenna to have sucked out cataracts through a hollow needle. How far they succeeded I am not able to state.

In 1847, M. Langier invented his "aiguille a pompe," a hollow needle fixed in a syringe, apparently like that now in use for subcutaneous injection. Its use is discussed by M. Desmarres. The needle having been thrust through the sclerotica, vitreous humour, and posterior capsule, and lodged in the centre of the lens, the suction was applied by means of the syringe in the handle of the instrument. If the cataract was fluid, it was drawn into the instrument, the pupil became clear, and sight was immediately restored. If the cataract were not fluid, ("et la cataracte est rare,") the vitreous humour was drawn out, the cataract was left *in situ*, and the eye collapsed. This misadventure was followed by internal inflammation of the eye, and in consequence, the operation was condemned. "En resume, l'operation de la cataracte par succion est abandonnée." Failure in this operation was to be expected from using the needle of the syringe as the piercing instrument, and from traversing the sclerotica and rupturing the posterior capsule, which ought to have been preserved as the barrier between the cataract and vitreous humour.

Again, M. Blanchet brought forward another method of extracting cataract by suction. Having dilated the pupil, he made an opening in the cornea with a broad needle, through which he introduced a small tube with a flageolet-like mouth attached to an Anell's syringe. With this blunt tube he pierced the capsule of the lens; and if the cataract proved soft, he pumped it out through the tube by working the piston of the syringe. The main defects of this plan of M. Blanchet, were—first, the attempt to puncture the anterior capsule with a blunt instrument, thereby using unnecessary force in reaching the cataract; and secondly, the imperfect opening of the anterior capsule, whereby the capsule remained in the area of the pupil, and becoming opaque, rendered a secondary operation necessary.

*On Suction Instruments.*—The original suction instrument which M. Weiss made for me consisted simply of a tubular curette fixed in a handle, to which a small india-rubber tube with a mouth-piece is attached. The flexible tube is of such a length as to reach from the mouth of the operator to the curette when held in the eye.

Shortly afterwards, M. Weiss made, at the suggestion of Mr. Bowman, a modification of this instrument, in which the suction power is applied by an ingenious mechanism in the handle, so that the hand which holds the curette controls the suction. Mr. Bowman, also, inserted a piece of glass tube between the curette and the handle, to enable the operator to watch the result of the suction.

A third instrument has been suggested and made for me by M. Weiss, which is simply a light glass tube with the tubular curette fixed at one end, and the flexible tube with the mouth-piece, at the other end.

A fourth instrument has been made for Dr. Badu, of Guy's hospital, by Khrone, of Whitechappel, and is thus described by Mr. Lawson: "The suction-power is a small, hollow, india-rubber ball, placed at the extremity of a tnbe which terminates in a glass tubular curette. Pressure is made on the ball with the hand to expel the air from the tube, and its re-admission is regulated by a well-contrived stop apparatus placed close to the curette. After the air from the ball has been expelled and its re-admission prevented by closing the stop the curette is introduced into the eye, and the amount of suction is regulated by a little trigger connected with the stop apparatus within."

Having used the first three forms of the instrument, I have found them to do their work perfectly; and I have no doubt that Dr. Badu's is at least equal to them. On the whole, perhaps from having used it more frequently, I prefer the original curette, (with the addition of the glass tube,) as the suction is more immediately at command when applied by the mouth, and the instrument can be guided with greater delicacy when the hand is not fettered by applying the suction power.

[Dr. Teale reports seven cases operated on by this method. The details are omitted; but they were all successful, and unfollowed, except in an instance—that of a scrofulous child—by any serious inflammation ]

